# Strain-induced antipolar phase in hafnia stabilizes robust thin-film ferroelectricity

**DOI:** 10.1126/sciadv.add5953

**Published:** 2022-11-25

**Authors:** Songsong Zhou, Jiahao Zhang, Andrew M. Rappe

**Affiliations:** Department of Chemistry, University of Pennsylvania, Philadelphia, PA 19104-6323, USA.

## Abstract

Hafnia (HfO_2_) is a promising candidate for next-generation ferroelectric devices due to its robust ferroelectricity at reduced dimensions and its compatibility with silicon technology. Unfortunately, the origin of robust ferroelectricity and the underlying phase transition mechanism in HfO_2_ remain elusive. Here, we show that its ferroelectricity arises from two phase transitions, where the primary phase transition to antipolar phase is activated by tensile strain. Above a threshold antipolar mode amplitude, a strong cooperative polar-antipolar coupling enables a second ferroelectric phase transition superimposed on the antipolar phase. Because the antipolar mode is not susceptible to depolarization, this polar-antipolar coupling stabilizes the polarization against depolarization effects. Our results demonstrate that tensile strain and polar-antipolar coupling are the origins of ferroelectricity in HfO_2_ and provide a previously unknown mechanism against depolarization other than conventional improper ferroelectricity.

## Teaser

Strain induces two successive phase transitions that stabilize the robust ferroelectricity down to the thinnest HfO_2_ films.

## INTRODUCTION

Ferroelectrics have spontaneous electric polarization that can be switched to different crystallographic directions under an electric field. Ultrathin ferroelectrics are of great technological interest for miniaturizing semiconducting devices, such as ferroelectric memories, neuromorphic circuits, and negative differential capacitance transistors (*1*–*3*). However, as material size is reduced to the nanometer scale, the ferroelectricity of conventional perovskite oxides is suppressed because of the depolarizing field arising from the surface charges (*4*, *5*). Bulk hafnia (HfO_2_) forms in the equilibrium cubic fluorite ($\mathrm{Fm}\bar{3}m$), tetragonal (*P*4_2_/*nmc*), and monoclinic baddeleyite (*P*2_1_/*c*) phases at atmospheric pressure, all of which are centrosymmetric. Recently, the discovery of ferroelectricity in thin films of orthorhombic (*Pca*2_1_) HfO_2_, which is absent in the bulk equilibrium phase diagram, provides a new avenue for achieving thin ferroelectric devices (*1*, *6*–*9*). Their robust ferroelectricity at reduced dimensions and compatibility with current silicon technologies make HfO_2_ a promising candidate for next-generation devices (*6*, *10*, *11*). Various external factors such as surface energy (*12*, *13*), vacancies and dopants (*14*–*19*) and strain (*20*, *21*) have been proposed to stabilize the ferroelectric phase. The role of strain is highlighted by the recent finding that the thin films exhibit ferroelectric signatures only in the regions where strain is applied (*10*). In this work (*10*), a large value of aspect ratio *R*_aspect_ ≡ 2*a*/(*c* + *b*) > 1.1 [*a* is the tetragonal (longest) axis] has also been measured, whereas the bulk orthorhombic equilibrium value is only about 1.02 to 1.04. This unusually large aspect ratio indicates that the thin film experiences substantial elongation along the tetragonal axis. Such strain state is also consistent with the recent report of the domain matching epitaxy growth mechanism in Hf_0.5_Zr_0.5_O_2_ (*22*). Through this mechanism, large misfit is accommodated by matching different integer numbers of atomic planes of the substrate (La_2/3_Sr_1/3_MnO_3_) and the thin film, leading to about 3.5% tensile strain (when referenced to orthorhombic phase) in the tetragonal direction (*23*). Moreover, upon rapid thermal annealing with a capping layer, the asymmetric thermal expansion (α*_a_* > α_*b*/*c*_) leads to asymmetric strain response, mainly elongation of the tetragonal direction (*24*, *25*). These sources of uniaxial tensile strain along the tetragonal axis would also be most advantageous for the stabilization of the orthorhombic phase (*21*). The phase transition has been proposed to be an improper ferroelectric phase transition, which would not have a critical thickness and could therefore explain the robustness of ferroelectricity in thin films (*26*, *27*). However, the linear coupling of the proposed primary order parameter and polarization is forbidden by symmetry for HfO_2_ (*28*–*31*). Biaxial in-plane epitaxial tensile strain along the short axes of the tetragonal phase is reported to lead to structural instability of the tetragonal phase and thus induce a phase transition involving simultaneous freezing in of antipolar mode and polar mode (*20*), which highlights the critical role of strain in ferroelectric phase. However, under this proposed orientation and strain state, the polarization would lie in the substrate surface and thus lead to almost zero remanent polarization out of plane. Moreover, such strain state tends to decrease the orthorhombic aspect ratio *R*_aspect_, which is measured to be very large, ≈1.1 in experiments (*10*). A one-step transition mechanism involving primary polarization instability and an additional strain-induced instability has been proposed to explain the phase transition, yet the leading order parameter and the origin of robust ferroelectricity are unclear (*23*). In summary, the understanding of the phase transition mechanism is still limited, and why ferroelectricity is robust in HfO_2_ thin film is still unknown.

Here, we use first-principles calculations to demonstrate that ferroelectricity in orthorhombic HfO_2_ originates from two successive, interrelated phase transitions, and the robustness derives from the strong, cooperative (energy-lowering) polar-antipolar coupling. We propose that, unlike ordinary proper ferroelectrics, the primary instability of tetragonal HfO_2_ is not the polar mode but an antipolar mode, which is induced by tensile strain. As the result of this first antipolar (AP) phase transition, the antipolar mode freezes in. We find that the antipolar mode amplitude cooperatively couples to polarization. Because of this strong and cooperative polar-antipolar coupling, the finite antipolar amplitude acts to stabilize structures that also have finite polarization, inducing the ferroelectric (FE) phase transition to form an AP + FE phase with its unique alternating pattern of polar and nonpolar planes. Moreover, this coupling not only induces the ferroelectric transition but also stabilizes the electric polarization against depolarization field at reduced dimensions. Last, we show that tensile strain can fundamentally change the polarization switching mechanism in HfO_2_ and could explain the thin-film ferroelectric stability. Our work resolves mysteries about the origin of robust ferroelectricity and the phase transition mechanism in HfO_2_ and suggests manipulating strain as a strategy for controlling its properties.

## RESULTS AND DISCUSSION

**Fig. 1.**
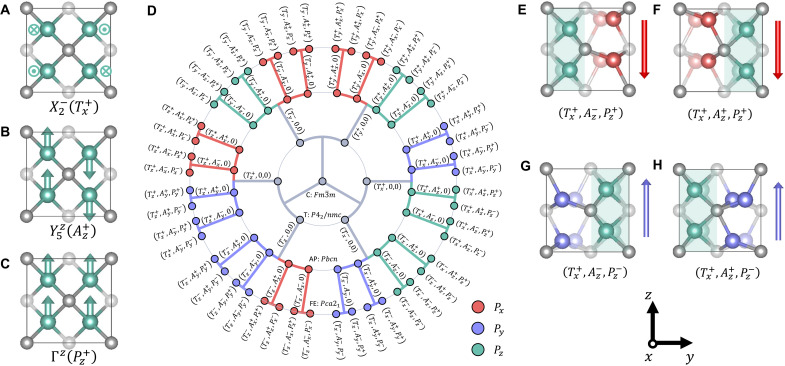
Succession of phase transitions in HfO_2_. The structural change can be indexed by three modes—tetragonal mode ($T_{\alpha }^{n}$), antipolar mode ($A_{\beta }^{n^{\prime }}$), and polar mode ($P_{\beta }^{n^{\prime \prime }}$), where the subscript and superscript of each component denote the mode direction (α = *x*, *y*, and *z* with β ≠ α) and sign of mode amplitude (*n*, *n*′, and *n*″ = ±), respectively. Without loss of generality, we illustrate, for the α = *x* and β = *z* case, (**A**) $X_{2}^{-}$, the tetragonal mode denoted by $T_{x}^{+}$, where oxygen atoms are displaced in an antiparallel pattern in (011) planes; (**B**) $Y_{5}^{z}$, the antipolar mode denoted by $A_{z}^{+}$, where oxygen atoms are displaced along the *z* direction in an antipolar pattern; and (**C**) Γ*^z^*, the polar mode denoted by $P_{z}^{+}$, where all oxygen atoms move in the *z* direction. The arrows denote the oxygen atom displacements. (**D**) Starting from the cubic phase in the center, condensing tetragonal, antipolar, and polar modes sequentially will generate 6, 24, and 48 variants of the tetragonal, antipolar, and ferroelectric (AP + FE) orthorhombic phases, respectively. Each variant ($T_{\alpha }^{n}$, $A_{\beta }^{n^{\prime }}$, $P_{\beta }^{n^{\prime \prime }}$) is represented by a node colored according to the direction of polarization. (**E** to **H**) Example of four variants $\left(T_{x}^{+},A_{z}^{\pm },P_{z}^{\pm }\right)$. The near-exact cancellation of polar and antipolar mode displacements in half the unit cells leads to nonpolar layers (green shading). The signs of the *A_z_* and *P_z_* modes determine the polarization direction and the nonpolar layer position. Gray, green, red, and blue spheres denote the Hf atoms, nonpolar oxygen atoms, and down- and up-polarized oxygen atoms, respectively. The transparency of spheres denotes distance from the viewer.

To examine the unusual polar-antipolar mode coupling, we analyze the sequence of phase transitions and the associated phonon modes. The phase transitions from cubic to tetragonal to orthorhombic phase could be fully described by eight phonon modes (the Supplementary Materials), while three of them are most important—tetragonal mode ($T_{\alpha }^{n}$), antipolar mode ($A_{\beta }^{n^{\prime }}$), and polar mode ($P_{\beta }^{n^{\prime \prime }}$) (Fig. 1, A to C) (*32*, *33*). The superscript and subscript denote the sign of amplitude and direction of atomic displacement, respectively. Note that β ≠ α so that polar axis is perpendicular to the long tetragonal axis. Given the amplitudes of these three modes, the amplitudes of the other five modes could be determined correspondingly during relaxation. The structure of HfO_2_ could then be defined by a mode vector $\left(T_{\alpha }^{n},A_{\beta }^{n^{\prime }},P_{\beta }^{n^{\prime \prime }}\right)$. The corresponding phase transitions in HfO_2_ are portrayed in a radial diagram (Fig. 1D). Upon cooling, the cubic phase ($\mathrm{Fm}\bar{3}m$) in the center of the radial diagram can convert to any of six tetragonal variants (*P*4_2_/*nmc*) in the first inner circle by condensing the tetragonal mode along different directions (*34*). Next, condensing an antipolar mode will generate 24 variants of the intermediate antipolar phase (*Pbcn*) by choosing a displacement direction and phase of the *A* mode. Last, condensing a polar mode with approximately the same amplitude as the *A* mode generates 48 variants of the ferroelectric (AP + FE) orthorhombic phase (*Pca*2_1_) in the outer circle. The mixing of polar and antipolar modes leads to the destructive superposition of atomic displacement of half the atoms and constructive superposition of the other half (*26*). Consequently, an unusual pattern of alternating polar/nonpolar layers is generated in the (AP + FE) phase (Fig. 1, E to H).

**Fig. 2.**
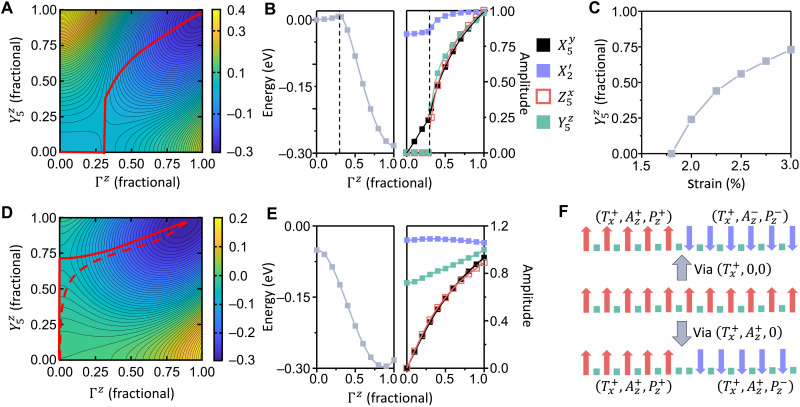
Strain stabilization of antipolar and ferroelectric phases of HfO_2_. (**A**) The energy of HfO_2_ as a function of polar and antipolar modes under zero strain (tetragonal lattice parameters; all other modes are relaxed) shows that the tetragonal phase is metastable. (The mode amplitudes are normalized to the equilibrium orthorhombic phase.) (**B**) As the polar mode amplitude is increased [at zero strain; see red solid line in (A)], energy rises until an energy cusp [saddle point in (A)] is reached, beyond which large antipolar and polar mode amplitudes coexist. (**C**) For tensile strain ε*_xx_* of > 1.9%, the tetragonal phase is no longer metastable, and the equilibrium antipolar mode amplitude at Γ*^z^* = 0 is shown. (**D**) Under 3% tensile strain, the lowest-energy path from tetragonal to orthorhombic phase (red dashed line) is shown. The antipolar mode is unstable even at *P* = 0 (leading to the *Pbcn* phase), and after the antipolar mode amplitude increases sufficiently, the polar mode then becomes unstable, leading to the orthorhombic AP + FE phase. (**E**) As the polar mode amplitude is increased from zero (at 3% tensile strain), the energy decreases to the equilibrium AP + FE phase. (**F**) Switching an up-polarized orthorhombic variant $\left(T_{x}^{+},A_{z}^{+},P_{z}^{+}\right)$ to the down-polarized variants $\left(T_{x}^{+},A_{z}^{+},P_{z}^{-}\right)$ or $\left(T_{x}^{+},A_{z}^{-},P_{z}^{-}\right)$ could generate domain walls consisting of two (fast-switching domain wall) or one (slow-switching domain wall) nonpolar layer(s), respectively. These two switching paths will pass through the *Pbcn* and the tetragonal phases, respectively. The second switching path is not energetically favorable, because the stability of antipolar mode requires additional energy to switch antipolar and polar mode together.

This unique pattern of polar and nonpolar layers underscores the importance of cooperative coupling between the polar and antipolar modes. To explore this coupling, we first calculate the energy of HfO_2_ as a function of fixed polar and antipolar modes under zero strain (Fig. 2A). The initial amplitude of the tetragonal mode is set to its equilibrium value in the tetragonal phase, and the initial amplitudes of the other five modes are set to be zero. All the modes except for the fixed polar and antipolar mode amplitudes are fully relaxed to search the entire phase space. Without losing generality, we set the polar (*c*) axis and tetragonal (*a*) axis to the *z* and *x* directions, respectively. Accordingly, the polar mode and antipolar mode are Γ*^z^* and $Y_{5}^{z}$, respectively. As reported in recent zero-stress calculations, the tetragonal phase is metastable (*33*). Increasing the amplitude of the Γ*^z^* or $Y_{5}^{z}$ mode alone increases the energy. However, by mixing the two modes together, the energy could be lowered if the amplitudes are larger than ≈0.3 (all amplitudes are normalized to their values in the equilibrium orthorhombic phase). This energy drop indicates that the polar-antipolar coupling has an unusual negative coefficient, in contrast to the positive (energy-raising) coupling between the two modes in conventional ferroelectrics. Such coupling could be further confirmed by considering the energy as a function of polar mode amplitude (Fig. 2B). For small values of Γ*^z^*, the energy increases with respect to the polarization, while the $Y_{5}^{z}$ remains stabilized at zero. At the critical value of Γ*^z^* ≈ 0.3, an energy cusp is found, where the $Y_{5}^{z}$ mode abruptly freezes in to a notable nonzero value. This cusp corresponds to a first-order phase transition where the constrained polar structure (*Aba2* phase) converts to the *Pca*2_1_ space group. At this cusp, the amplitude of the polar mode is large enough that the energy drop due to the polar-antipolar coupling offsets the energy increase due to the finite antipolar mode amplitude. Beyond this point, the polar-antipolar coupling stabilizes the ferroelectric AP + FE *Pca*2_1_ phase.

Next, we examine the role of tensile strain in enhancing the antipolar-polar mode coupling and its influence on phase stability. Under zero strain, the polar and antipolar modes will not freeze in spontaneously to induce phase transitions. However, by applying tensile strain along the tetragonal (*a*) axis, the antipolar mode loses its stability while the polar mode remains stable. A critical value of $\varepsilon_{\mathrm{xx}}=\Delta a/a_{\text{tetra}}^{0}>1.9\%$ (where $a_{\text{tetra}}^{0}$ is the equilibrium lattice constant along the tetragonal axis in the tetragonal phase) is required to induce this antipolar instability (Fig. 2C). This is much lower than the experimentally observed strain (*22*, *23*). As an example, we repeat the energy calculation with respect to Γ*^z^* and $Y_{5}^{z}$ under ε*_xx_* = 3%. The energy peak at the top left corner disappears because of the tensile strain. At fixed *P* = 0, $Y_{5}^{z}$ will freeze in to 0.72, forming the *Pbcn* phase (*35*) and lowering the energy by 0.013 eV/formula unit with respect to the tetragonal phase (Fig. 2D). Such strain-induced instability can be understood by considering lattice constant differences. Under zero stress, the high-energy *Pbcn* phase prefers a larger *x* lattice constant than the tetragonal phase. Tensile strain lowers the energy difference between the tetragonal and *Pbcn* phases, and above the critical strain, the *Pbcn* phase becomes lower in energy. Thus, tensile strain will stabilize the *Pbcn* phase and induce the instability of antipolar mode.

Once the antipolar mode freezes in with sufficient amplitude, the polar mode also becomes unstable. The energy decreases below the *Pbcn* phase energy as the polar mode amplitude increases (Fig. 2E), and a downhill energy path from tetragonal through *Pbcn* to orthorhombic phase can be found. This phase transition path with zero energy barrier could be further confirmed by density functional theory relaxation, showing that the tetragonal phase relaxes to orthorhombic phase under uniaxial tensile strain. Consequently, the transition from tetragonal phase to orthorhombic is a two-step transition. The second ferroelectric transition (to the AP + FE state) is triggered by the primary antipolar transition, whose instability is caused by tensile strain (*36*, *37*). This is unlike the mechanism of conventional AFE transition, where the freezing in of an antipolar mode will suppress the ferroelectric phase transition because of repulsive polar-antipolar mode coupling. In HfO_2_, however, freezing in of the antipolar mode facilitates the ferroelectric transition because of cooperative polar-antipolar coupling, thus leading to the coexistence of AP + FE state and the pattern of polar/nonpolar layers.

The strain-induced antipolar instability also changes the polarization switching mechanism. As shown in previous work (*38*), under zero strain, a down-polarized state $\left(T_{x}^{+},A_{z}^{+},P_{z}^{+}\right)$ could be switched to two up-polarized states, $\left(T_{x}^{+},A_{z}^{+},P_{z}^{-}\right)$ and $\left(T_{x}^{+},A_{z}^{-},P_{z}^{-}\right)$, located in the region $\left(Y_{5}^{z}>0,\Gamma^{z}<0\right)$ and $\left(Y_{5}^{z}<0,\Gamma^{z}<0\right)$, respectively. The energy landscape in these regions could be generated by applying mirror operation along *x* and/or *y* axis to the landscape in $\left(Y_{5}^{z}>0,\Gamma^{z}>0\right)$ of Fig. 2D. Both transition paths without strain pass through the tetragonal phase $\left(T_{x}^{+},0,0\right)$ and thus have same energy barrier. However, with the antipolar mode freezing in (due to strain above threshold), the *Pbcn* phase $\left(T_{x}^{+},A_{z}^{+},0\right)$ has lower energy than tetragonal phase. Thus, the transition (Fig. 2F) to $\left(T_{x}^{+},A_{z}^{+},P_{z}^{-}\right)$ now passes through the *Pbcn* phase instead of the tetragonal phase and involves a barrier of 0.25 eV per unit cell. On the other hand, the transition to $\left(T_{x}^{+},A_{z}^{-},P_{z}^{-}\right)$ still passes through the tetragonal phase and has higher energy barrier (≈0.30 eV per unit cell) because of the antipolar instability. The barriers and transition paths are confirmed by nudged elastic band calculations (the Supplementary Materials). Consequently, the $\left(T_{x}^{+},A_{z}^{+},P_{z}^{+}\right)$ can only be switched to $\left(T_{x}^{+},A_{z}^{+},P_{z}^{-}\right)$. This switching process will form a domain wall consisting of two consecutive nonpolar layers, while the other transition path will form domain walls with only one nonpolar layer. The domain wall of two nonpolar layers is believed to have much lower switching barrier and to allow the nucleation-and-growth switching mechanism for ultrafast polarization switching (*39*).

**Fig. 3.**
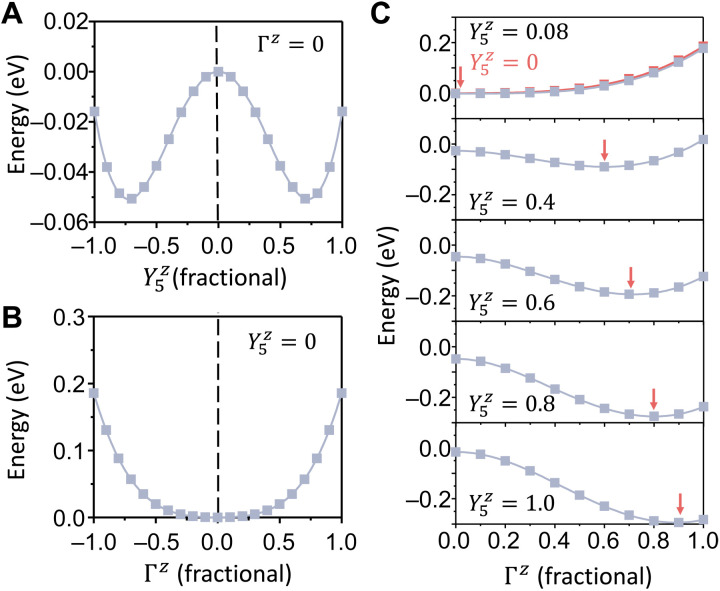
Primary antipolar transition and successive ferroelectric transition in HfO_2_ under 3% tensile strain. (**A**) At zero polar mode, the double-well potential with respect to antipolar mode is induced by tensile strain. (**B**) The polar mode vibration Γ*_z_* is still stable under zero antipolar mode $Y_{5}^{z}$. (**C**) Subsequently, as the antipolar mode amplitude freezes in, the polar mode is still stabilized at zero when antipolar mode is small. When the antipolar mode exceeds a threshold amplitude, then the polar mode instability is induced, and its amplitude increases as the antipolar mode amplitude increases further.

To further illustrate the phase transition mechanism, a Landau-Ginzburg-Devonshire model based on symmetry is proposed

$$\begin{array}{c} f(A,P,\varepsilon )=E_{0}+\kappa_{00}^{2}\varepsilon^{2}+(\kappa_{02}^{0}+\kappa_{02}^{1}\varepsilon +\kappa_{02}^{2}\varepsilon^{2})P^{2}+(\kappa_{04}^{0}+\kappa_{04}^{1}\varepsilon +\kappa_{04}^{2}\varepsilon^{2})P^{4}\\ +(\kappa_{2\;0}^{0}+\kappa_{2\;0}^{1}\varepsilon )A^{2}+(\kappa_{4\;0}^{0}+\kappa_{4\;0}^{1}\varepsilon )A^{4}+(\kappa_{2\;2}^{0}+\kappa_{2\;2}^{1}\varepsilon )A^{2}P^{2}\\ +(\kappa_{2\;4}^{0}+\kappa_{2\;4}^{1}\varepsilon +\kappa_{2\;4}^{2}\varepsilon^{2})A^{2}P^{4}+(\kappa_{4\;2}^{0}+\kappa_{4\;2}^{1}\varepsilon +\kappa_{4\;2}^{2}\varepsilon^{2})A^{4}P^{2} \end{array}$$ (1)

where *A* and *P* are the order parameters associated with antipolar and polar mode amplitudes, respectively. *E*_0_ is the energy of the tetragonal phase under zero strain. Under zero strain, the coefficients of quadratic terms of both *A* and *P* are positive. Thus, single-well potentials are formed that stabilize both *A* and *P* at zero. As tensile strain ε increases, the $\kappa_{20}^{\varepsilon }=\kappa_{20}^{0}+\kappa_{20}^{1}\varepsilon$ becomes negative and thus induces the antipolar phase transition, where *A* freezes to nonzero amplitude (Fig. 3A). The $\kappa_{02}^{\varepsilon }$ remains nonnegative, and thus the polarization is still stabilized at *P* = 0 when *A* = 0 (Fig. 3B). Through the coupling terms, the nonzero *A* renormalizes the coefficient of the *P*^2^ term to $\lambda_{02}^{\varepsilon }=\kappa_{02}^{\varepsilon }+(\kappa_{22}^{0}+\kappa_{22}^{1}\varepsilon )A^{2}+(\kappa_{42}^{0}+\kappa_{42}^{1}\varepsilon +\kappa_{42}^{2}\varepsilon^{2})A^{4}$. For small values of *A*, the values of the last two terms are still smaller than $\kappa_{02}^{\varepsilon }$ so that $\lambda_{02}^{\varepsilon }$ remains positive, and *P* is still stabilized at zero (Fig. 3C). With increasing *A* amplitude, $\lambda_{02}^{\varepsilon }$ becomes negative, and then the *P* instability is induced. Such a two-step phase transition mechanism is similar to but not the same as an improper ferroelectric phase transition (*30*), where the primary instability induces the secondary polarization instability simultaneously. In an improper ferroelectric transition, the primary order parameter couples linearly to polarization (*28*); the primary order parameter thus acts like an external field that pushes the center of the single-well potential off zero to induce spontaneous polarization (*27*), and as the primary order parameter freezes in, the secondary order parameter *P* should freeze in simultaneously. However, in the AP phase of HfO_2_ with $A_{z}^{+}$, either $P_{z}^{+}$ or $P_{z}^{-}$ can freeze in, generating degenerate orthorhombic variants related by inversion symmetry, as shown in Fig. 1 (F and H). Thus, the potential versus *P* with asymmetric single-well form is forbidden by symmetry. Consequently, *P* is not a secondary order parameter but the primary order parameter of a subsequent proper phase transition. Unlike the linear coupling to polarization in an improper ferroelectric that acts like an external field to induce polarization, increasing quadratic coupling strength in HfO_2_ will change the sign of the *P*^2^ term, thus inducing a proper ferroelectric phase transition. The strength of the coupling term, which is mainly determined by the amplitude of antipolar mode, must reach a threshold value to induce spontaneous polarization. Thus, a lag effect exists between primary antipolar instability and secondary ferroelectric instability, as shown in Fig. 3C. This lag effect implies that, in principle, there must be a strain range where the *A* freezes in, but its amplitude is not large enough to induce the *P* instability. Within this range, the meta-stable antipolar *Pbcn* phase could be stabilized.

The cooperative coupling between *A* and *P* answers the long-standing question about why the ferroelectricity in HfO_2_ is robust even in thin film. In a thin film, the uncompensated depolarization field is proportional to the polarization, *E_d_* = −λ*_d_P*. This adds a positive energy term to Landau polynomial, *f_d_* = +λ*_d_P*^2^ (*40*, *41*). In prototypical perovskite ferroelectric oxides, such as PbTiO_3_, the λ*_d_* ≈ 6.4 × 10^9^ J·m/C^2^ is sufficient to overcome the sign of the *P*^2^ bulk coefficient (≈ −5.2 × 10^8^ J·m/C^2^) and thereby suppress the spontaneous polarization. In HfO_2_, however, the strong coupling to the antipolar mode adds a huge negative energy term quadratic in *P*. This coupling could be considered as a polarizing field to offset the depolarization effect. We estimate that the bulk coefficient of the *P*^2^ term in HfO_2_ (≈ −6.9 × 10^9^ J·m/C^2^) is one order of magnitude larger than that of PbTiO_3_ and is about 1.6 times as large as the fully uncompensated depolarization field effect of HfO_2_ (λ*_d_* ≈ 4.2 × 10^9^ J·m/C^2^). Consequently, even the completely uncompensated depolarization field cannot suppress the polarization in HfO_2_ down to the thinnest films. This is a previously unknown mechanism against depolarization effect other than the conventional improper ferroelectricity. On the other hand, such strong polar-antipolar coupling not only offsets the depolarization field but also increases the coercive field. To enable easier polarization switching, one could control the strain to decrease the amplitude of the antipolar mode, which would lower the polar-antipolar coupling effect and the coercive field.

The crucial role of tensile strain in the primary antipolar transition may also explain the stabilization of HfO_2_ in thin films, the unusual reverse size effect (*10*). The strain is generally imposed via interfaces in thin films. The strain distortion decays with respect to the distance from the interface. Thus, in thick films, the strain distortion may not be sufficient to induce the primary antipolar transition. Consequently, the HfO_2_ in regions with insufficient strain distortion could be stabilized in the tetragonal phase. On the other hand, as the thickness decreases, the strain effects become larger, which favors antipolar transition and increases the amplitude of the antipolar mode. The antipolar mode tends to increase the $\lambda_{02}^{\varepsilon }$, which could offset the depolarization effect and thus increase the remanent polarization. This may contribute to the reverse size effect observed in experiments. Last, the tensile strain may also prevent the HfO_2_ thin film from being in a nonpolar phase, and more of the material will convert to single-phase polar orthorhombic. Because the presence of the nonpolar phase reduces the remnant polarization, increased strain can make the wake-up effect weaker. In summary, we demonstrate that the ferroelectricity in HfO_2_ originates from an unconventional cooperative polar-antipolar coupling and a tetragonal to antipolar to AP + FE orthorhombic phase transition mechanism. The tensile strain is critical to induce the primary instability of the antipolar transition, while the second ferroelectric transition is enabled by the cooperative polar-antipolar coupling. Our work reveals the origin of robust ferroelectricity and the mechanism of ferroelectric phase transition in HfO_2_. From a theoretical perspective, we provide insight into the multistep ferroelectric phase transition mechanisms and highlight the importance of cooperative coupling between polarization and other structural distortions. From a technological perspective, we also illustrate how the strain effect could induce and stabilize the ferroelectric phase. Moreover, the strain-induced antipolar instability confers stability of the ferroelectric phase down to the thinnest film thicknesses and also changes the polarization switching path, which favors the specific domain wall structure that leads to fast polarization switching. Our results suggest accurately manipulating strain as a strategy to control the polarization switching and to improve the performance of HfO_2_-based thin-film devices.

## MATERIALS AND METHODS

Density functional theory calculations were performed using the Vienna Ab initio Simulation Package (VASP) (*42*, *43*) with a plane-wave basis set and the projector augmented-wave method (*44*, *45*). The strongly constrained and appropriately normed functional was adopted to describe the exchange correlation energy functional (*46*). The plane-wave cutoff energy was set to 500 eV, and the Brillouin zone integrals were sampled by a 4 by 4 by 4 *k*-point mesh. All structures were fully relaxed until the total force on each atom is smaller than 0.01 eV/Å. To calculate the energy under fixed polar (Γ*^z^*) and antipolar mode ($Y_{5}^{z}$) amplitudes, the coordinates of atoms along the *z* axis were fixed. The other six modes whose atomic displacement is along *x* or *y* axis were fully relaxed under constraint of fixed amplitudes of Γ*^z^* and $Y_{5}^{z}$. The initial amplitude of each mode, except for tetragonal, polar, and antipolar modes, is set to be zero. The space groups of various crystal structures are identified by the FINDSYM program (*47*). To simulate the uniaxial tensile strain along *x* axis, the lattice constants along the *y* and *z* axes were fixed at the unstrained value of the tetragonal phase. The fractional amplitude of each mode was normalized with respect to its corresponding amplitude in the equilibrium orthorhombic phase. To fit the coefficients in Eq. 1, high weight was given to the data points along the *A* and *P* axes to ensure correct description of the instability/stability of order parameters. The uncompensated depolarization effect was estimated by λ*_d_* = −1/ε_ferro_ε_0_, where ε_ferro_ and ε_0_ are the dielectric constant of ferroelectric materials and vacuum permittivity, respectively (*48*). The coefficient of the quadratic term of polarization was estimated by fitting several additional points of energies versus polarization near *P* = 0 (note that for HfO_2_, the *A* is fully relaxed). The polarization value of each structure is calculated by the Berry’s phase method (*49*, *50*). The figures of modes and atomic structures are plotted by the VESTA code (*51*). The polarization, dielectric constant, and bandgap of orthorhombic phase as a function of strain are summarized in table S2. These values match with experimental measurement (*9*, *52*). Phonon dispersion was calculated by VASP using a 2 by 4 by 4 supercell and postprocessed by Phonopy code (*53*).

## Supplementary Materials

### This PDF file includes:

Supplementary Text Sections S1 to S5 Figs. S1 to S7 Tables S1 and S2
