# Supplementary material for: Strain-induced antipolar phase in hafnia stabilizes robust thin-film ferroelectricity
Source: Sci Adv. 2022 Nov 25;8(47):eadd5953. doi: 10.1126/sciadv.add5953 (PMC9699663; doi:10.1126/sciadv.add5953)
Supplement: Supplementary file 1 — Supplementary Text Sections S1 to S5 Figs. S1 to S7 Tables S1 and S2 [file sciadv.add5953_sm.pdf]

Science Advances
AAAS

Supplementary Materials for

Strain-induced antipolar phase in hafnia stabilizes robust thin-film ferroelectricity

Songsong Zhou *et al.*

Corresponding author: Andrew M. Rappe, rappe@sas.upenn.edu

Sci. Adv. **8**, eadd5953 (2022)
DOI: 10.1126/sciadv.add5953

This PDF file includes:

Supplementary Text
Sections S1 to S5
Figs. S1 to S7
Tables S1 and S2

Supplementary Text

Section 1: Phonon modes to describe structural distortion

Using the phonon modes of the high-symmetry cubic phase as basis, the structural distortions of the orthorhombic phase can be decomposed into eight modes. In addition to the most important X_2^-, Γ^z, and Y_5^z modes, five additional modes are required to describe the structural distortions (Figure.S1 A-E). X_5^y and Z_5^x modes are degenerate with the antipolar Y_5^z mode, while their oxygen atomic displacements are along the y- and x-axis, respectively. Similar to X_5^y mode, the oxygen displacements in the X_5^{y-} mode is also along the y-axis. To distinguish these two modes, the oxygen atoms of same displacement in X_5^{y-} mode are along lines parallel to z-axis, while the oxygen atoms of same displacement in the X_5^y mode are along the yz planes. The large/small separation of oxygen atom layers in polar/nonpolar layers (Figure S1 F, $l_1 > l_2$) result from the constructive/destructive interference of the X_5^{y-} and X_5^y modes. Similarly, $s_1 > s_2$ in Figure S1 G results from the interference of X_2^- and Z_5^x mode. The last two modes involve the displacement of Hf atoms. The X_3^{y-} mode leads to the larger separation of Hf atom layers in spacer layers than that in nonpolar layer (Figure S1 F, $d_1 > d_2$). The X_5^{y-} and Y_5^{x-} mode condense simultaneously with Y_5^z mode to form the Pbcn phase (Figure 2 in the main text), while the X_5^y mode always condenses together with the Γ^z mode.

Section 2: Primary order parameter of successive phase transitions

In the basis of the cubic phase modes, eight modes are required to condense to form the orthorhombic phase. Upon cooling, the X_2^- mode freezes in to form the tetragonal phase and thus change the space group symmetry. In the tetragonal phase, three modes are involved in the transition from tetragonal to orthorhombic phase. They are P_T (involving Γ^z and X_5^y), M_T (involving Z_5^x and Y_3^-) and A_T (involving Y_5^z, Z_5^{x-} and X_5^{y-}). By condensing A_T, which involves the original Y_5^z mode, the tetragonal phase is transformed to intermediate $Pbcn$ phase. And from $Pbcn$ to orthorhombic phase, only a polar mode P_I (involves P_T and M_T) freeze

in. Consequently, the second step transition is a proper ferroelectric transition whose primary order parameter is polarization and the primary order parameter of the first step transition is the antipolar mode.

Section 3: Search of intermediate state in phase space

To further confirm that our relaxation calculation indeed find the only intermediate state before ferroelectric phase transition, we manually search the entire phase space. We consider the relaxation of structures of different initial value of each mode. Here, we start from tetragonal phase ($X_2^- > 0$), with P=0 (before ferroelectric transition), and then examine the relaxation of a series fixed value of A ($0 < A \leq 1$). For each fixed value of A, a series of initial configurations are generated with finite amplitude of the other five modes. These five modes are allowed to relax freely, and their initial value is generated randomly between 0 and 1.5, instead of zero in our original calculation in the manuscript. There are 100 different initial configurations randomly generated for each specific value of A, and in total 500 configurations are considered. It turns out that for each value of A, all configurations relaxed back to the same structure as our original relaxation. To visualize the result, for each fixed A amplitude, the relaxation result among 100 configurations that maximizes the energy difference w.r.t original relaxation result (Max($\Delta E = E_0 - E_i$), where E_0 and E_i are the energy of initial relaxation and current relaxation, respectively.) is plotted as red squares in Figure S4, in comparison with the original result E_0 as black squares. It could be seen in Figure S4 that black and red square match perfectly. Thus, we could confirm that all these structures should be relaxed to $Pbcn$, which is the only intermediate state.

Section 4: Effect of strain on the Phonon dispersion of tetragonal phase

Phonon dispersion at 0K was calculated to check the stability of all these modes under strain, as shown in Figure S5. All the relevant modes involved in the formation of orthorhombic phase are located at the high symmetry Γ point and M point of tetragonal phase. Under no

strain, tetragonal phase is metastable. Among these 8 modes, only the modes that form the $Pbcn$ phase show instability at the M point under 3% strain. Other modes are stable even in 0 K. Thus, under finite temperature, the other modes would still be stable and will not lead to other intermediate phase. This confirms our DFT calculation that $Pbcn$ is the intermediate state if strain is larger than threshold.

Section 5: Switching path between orthorhombic variants of different polarized state

In Figure 2D of main text, the energy landscape in the region ($Y_5^z > 0, \Gamma^z > 0$) is plotted, where the variant (T_x^+, A_z^+, P_z^+) is located in top right corner. Based on symmetry, the energy landscape in other three regions could be generated through mirror operation along $x-$ or $y-$axis (Figure S6). The path from (T_x^+, A_z^+, P_z^+) to (T_x^+, A_z^+, P_z^-) (located in the region $Y_5^z > 0, \Gamma^z < 0$) should pass through the antipolar phase ($T_x^+, A_z^+, 0$) and has an energy barrier of about 0.25 eV/unit cell. On the other hand, the path from (T_x^+, A_z^+, P_z^+) to (T_x^+, A_z^-, P_z^-) (located in region $Y_5^z < 0, \Gamma^z < 0$) should pass through the tetragonal phase ($T_x^+, 0, 0$) (original point in Figure 2D of main text) and has an energy barrier of about 0.30 eV/unit cell. This could be confirmed by NEB calculation, as shown in Figure S7. The NEB result confirmed our prediction of switching path, barrier height and intermediate state.

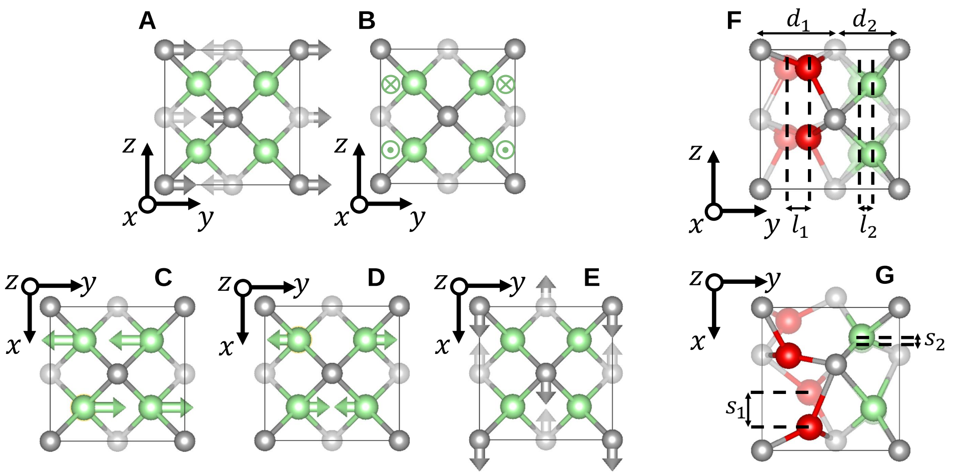

Figure S1: Phonon mode and corresponding atomic displacement. **(A)**Y_3^{-}, **(B)** Z_5^{x}, **(C)** X_5^{y}, **(D)**X_5^{y-} and **(E)**Z_5^{x-} mode. **(F)**, **(G)** The distance between polarized atoms in $y-$ and $x-$directions are larger than that of unpolarized atoms.

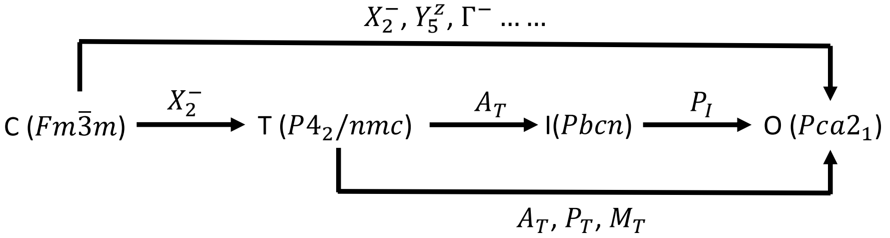

Figure S2: Phase transition from cubic (C) to tetragonal (T) to intermediate (I) to orthorhombic (O) phase and the order parameters.

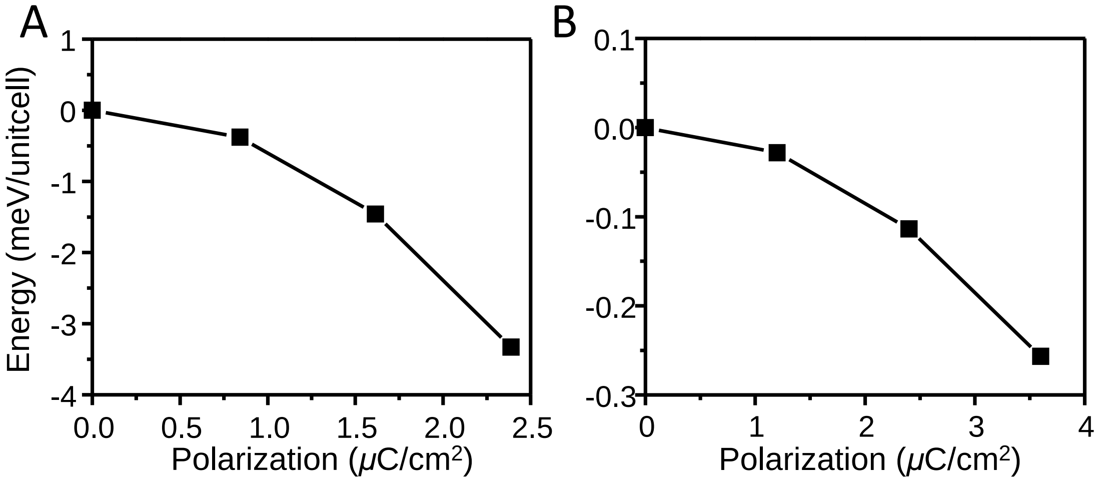

Figure S3: Estimation of coefficient of quadratic term of polarization by fitting energy *vs*. polarization near $P = 0$ for (A) HfO_2 with A fully relaxed and (B) $PbTiO_3$, respectively.

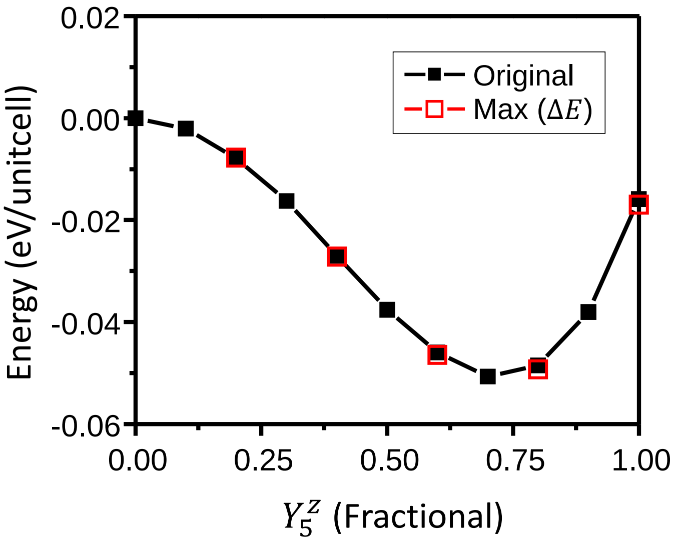

Figure S4: Comparison between the result of original relaxation and the result of manual searching through phase space of modes. The black square refers to the original relaxation result. The red square refers to the energy of relaxed configuration that maximize the energy difference ($\Delta E = E - E_0$, where E and E_0 are the energy of current relaxed result and the original result, respectively) w.r.t. the original result among 100 candidates.

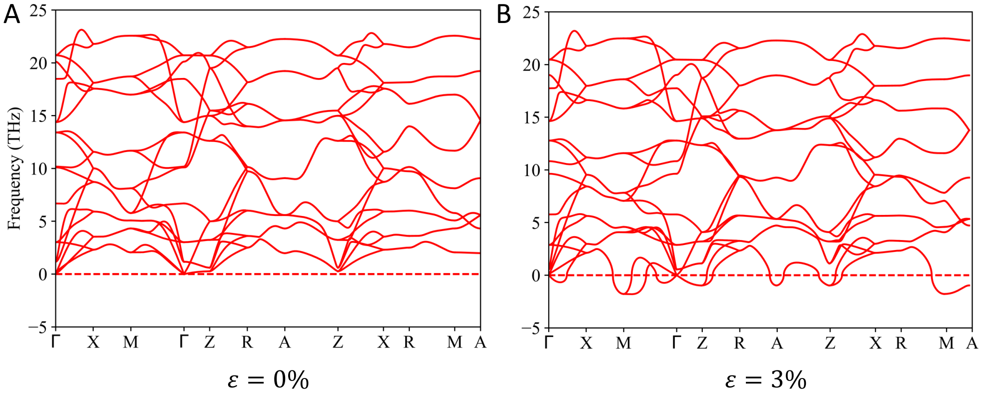

Figure S5: The phonon dispersion of tetragonal phase under strain (A) 0%, (B) 3%, respectively.

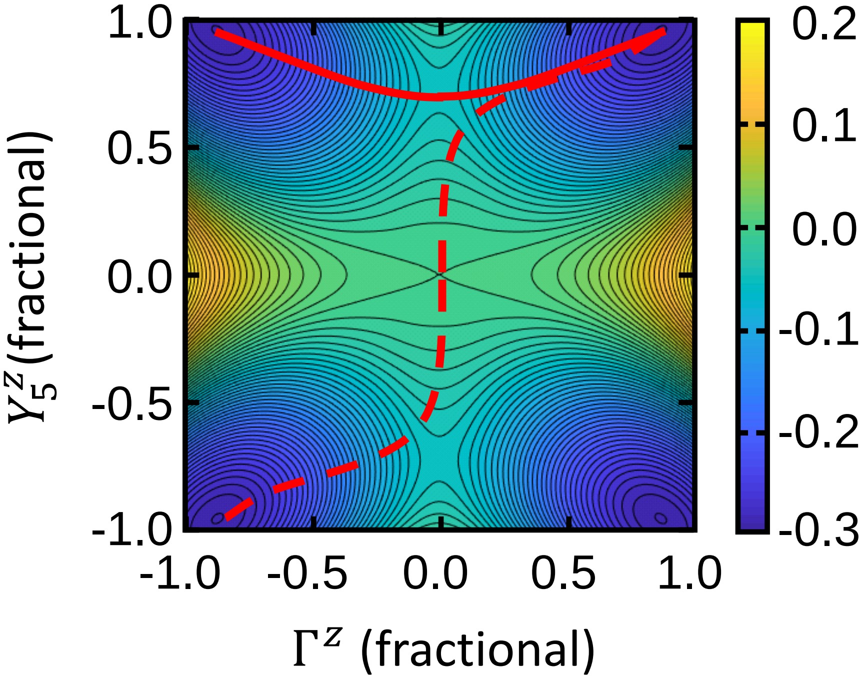

Figure S6: The energy landscape for $-1 < Y_5^z < 1$ and $-1 < \Gamma^z < 1$. The solid line and dash line correspond to the path from (T_x^+, A_z^+, P_z^+) to (T_x^+, A_z^+, P_z^-) and the path from (T_x^+, A_z^+, P_z^+) to (T_x^+, A_z^-, P_z^-), respectively.

Table S1: The parameter κ_{mn}^i (eV/unit cell) of polynomial term $(\sum \kappa_{mn}^i \varepsilon^i) A^m P^n$.

κ_{mn}^i		i		
m	n	0	1	2
0	0			107.42
2	0	0.352	-18.190	
4	0	0.082	3.262	
0	2	0.087	-2.760	44.166
2	2	-2.886	44.626	
4	2	0.836	-6.603	-37.303
0	4	0.140	0.416	-9.895
2	4	1.215	-25.898	123.261

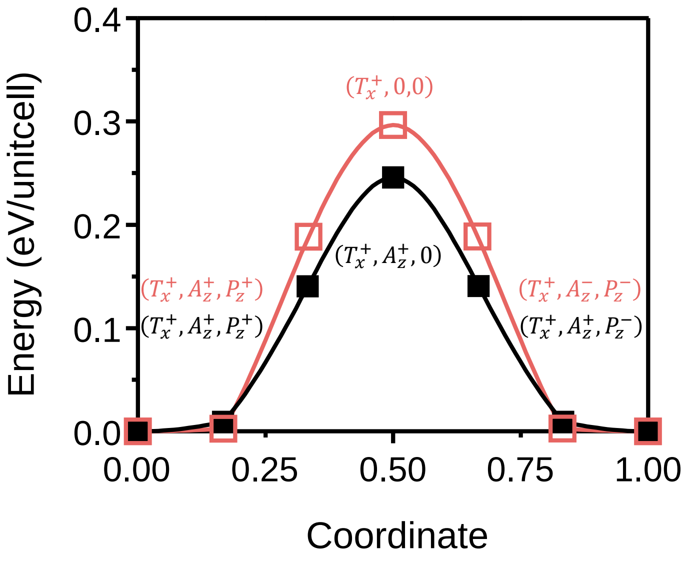

Figure S7: The NEB result of (black square) path from (T_x^+, A_z^+, P_z^+) to (T_x^+, A_z^+, P_z^-) and (red square) path from (T_x^+, A_z^+, P_z^+) to (T_x^+, A_z^-, P_z^-), respectively.

Table S2: The polarization (P), dielectric constant and bandgap as a function of strain.

Strain (%)	P (μC/cm^2)	Dielectric constant	Bandgap (eV)
0	53.31	22.30	4.92
1	50.22	23.20	4.97
2	46.94	24.70	4.99
3	42.89	26.75	5.04
Expt.	15-40[a]	25-27[b]	

a: Ref.52, polarization value depends on type of dopants.

b: Ref.9, temperature dependent, from 100 to 400K.
